# Quantitative ultrasound tissue characterization in shoulder and thigh muscles – a new approach

**DOI:** 10.1186/1471-2474-7-2

**Published:** 2006-01-18

**Authors:** Pernille Kofoed Nielsen, Bente R Jensen, Tron Darvann, Kurt Jørgensen, Merete Bakke

**Affiliations:** 1 Department of Physiology, National Institute of Occupational Health, Denmark; 2 Department of Human Physiology, Institute of Exercise and Sport Sciences, Faculty of Science, University of Copenhagen, Denmark; 3 3D Laboratory, Informatics and Mathematical Modelling, Technical University of Denmark, Copenhagen University Hospital, and School of Dentistry, Faculty of Health Sciences, University of Copenhagen, Denmark; 4 Section of Clinical Oral Physiology, School of Dentistry, Faculty of Health Sciences, University of Copenhagen, Denmark

## Abstract

**Background:**

The echogenicity patterns of ultrasound scans contain information of tissue composition in muscles. The aim was: (1) to develop a quantitative ultrasound image analysis to characterize tissue composition in terms of intensity and structure of the ultrasound images, and (2) to use the method for characterization of ultrasound images of the supraspinatus muscle, and the vastus lateralis muscle.

**Methods:**

Computerized texture analyses employing first-order and higher-order grey-scale statistics were developed to objectively characterize ultrasound images of m. supraspinatus and m. vastus lateralis from 9 healthy participants.

**Results:**

The mean grey-scale intensity was higher in the vastus lateralis muscle (p < 0.05) than in the supraspinatus muscle (average value of middle measuring site 51.4 compared to 35.0). Furthermore, the number of spatially connected and homogeneous regions (blobs) was higher in the vastus lateralis (p < 0.05) than in the supraspinatus (average for m. vastus lateralis: 0.092 mm^-2 ^and for m. supraspinatus: 0.016 mm^-2^).

**Conclusion:**

The higher intensity and the higher number of blobs in the vastus lateralis muscle indicates that the thigh muscle contained more non-contractile components than the supraspinatus muscle, and that the muscle was coarser. The image analyses supplemented each other and gave a more complete description of the tissue composition in the muscle than the mean grey-scale value alone.

## Background

Conventional real-time B-mode ultrasonography is a valuable tool for quantitative assessment of skeletal muscle thickness, and high positive correlations have been found between muscle thickness and muscle volume and strength [1], respectively. Further, different tissue types reflect ultrasound differently. Muscle tissue appears dark whereas fat and connective tissue are bright in ultrasound images [2-4]. Morphological changes and diversity regarding the distribution of contractile and non-contractile tissue has been identified in the human muscle due to age, exposure, training condition, miscellaneous diseases, oedema, and pathological alterations. Healthy muscles look dark with sharp, bright lines whereas muscles with different neuromuscular diseases like muscle dystrophy, myopathy or motor neuron disorders are brighter and more diffuse in the structure when evaluated by ultrasonography [2,3,5-8]. In elderly well-trained muscles, the components are depicted distinctly with dark muscle tissue and sharp bright lines of connective tissue, whereas young diseased muscles and elderly untrained muscles are more brightly speckled compared to young healthy muscles and elderly trained muscles [9,10]. Because invasive biopsy technique is not suitable as a diagnostic procedure for larger groups, there is an obvious need for non-invasive screening methods applicable for evaluation of whole muscles.

Ultrasonographic evaluation of the human muscles has been performed in studies on the quadriceps muscle, the arm, low-back, and masticatory muscles [6,11,12]. Most studies used a subjective evaluation in terms of a visual inspection of the images or measurements of the muscle thickness, and only a few studies have used quantitative ultrasound to characterize the various components of the muscle tissue [4,11,13-15]. The quantitative evaluation included the distribution of the grey-scale levels of the pixels in the images to describe tissue characteristics. However, muscles with very different muscle structure in terms of the spatial distributions determined by quantitative ultrasound image analysis could result in identical grey-scale intensity. Even if diseased muscles typically look diffused or unstructured, a well-trained muscle could be characterized by almost the same mean grey-scale value because of a distinct intramuscular tendon, e.g. in the supraspinatus muscle. Thus, supplementary analyses are needed to characterize muscles effectively. Therefore, we developed a method, a so-called "blob analysis", related to the higher order grey-scale statistics of the image, i.e. the structures. The blob analysis aims at detecting spatially connected and homogeneous (in terms of grey level) regions ( = blobs) in the image. The frequency distribution (histogram) of the size (area, mm^2^) and number (mm^-2^) of blobs detected characterize the image in terms of the amount and extension of spatial structures present. It has been hypothesized that various forms of musculoskeletal diseases and overload may facilitate formation of non-contractile tissue in muscles [16,17].

The aim of the present study was to develop a non-invasive method based on quantitative ultrasound image analysis to characterize tissue composition in terms of intensity and structure.

## Methods

### Participants

The right and left supraspinatus muscles, and the right vastus lateralis muscle were scanned in nine participants (7 females and 2 males) with the mean age of 35.8 (23–47) years. All participants except one were right-handed and all were without musculoskeletal disorders. None of them had performed physical exercise prior to the measurements. In addition, the same muscles were scanned in one right-handed female laboratory technician (56 years old) with severe work-related musculoskeletal symptoms in the right shoulder region related to the supraspinatus muscle. The local ethical committee approved the study, and all participants were fully informed about the protocol and procedures, before giving their consent.

### Procedure

Ultrasonography was performed with an ultrasound scanner (Diasonics VST Masters Series System, GE-Medical, Denmark: "Small parts") fitted with a 5 (3.5–7.0) MHz transducer (5.0 MHz 60R CLA Curved Linear Array HI-Density Probe, GE-Medical, Denmark). The digital recordings were stored on discs. The settings of the ultrasound system was standardized for all the participants and kept constant during all measurements to avoid potential changes in the images due to different setttings. We used a depth setting of 5 cm. Depth-gain compensation was built-in in the used scanner, meaning that the gain automatically was increased, while the depth increased to compensate for the loss through the tissue scanned. The acoustic signal received by the ultrasound transducer was converted into digital 8 bit intensity values making the ultrasound image. Each image consisted of pixels with a size of 0.13 mm × 0.13 mm = 0.017 mm^2^. As the approximate speed of sound through an average tissue is 1540 m/s and the transducer used was 5 MHz, the wavelength was approximately 0.3 mm [2,3,18]. During the ultrasound scanning, the participants were sitting upright in a chair with the upper arms relaxed and kept in a vertical position and with the forearms resting on the thighs. Contact gel was used for acoustic coupling and care was taken not to exert undue pressure on the imaged tissue, since hard pressure from the transducer may influence the grey scale intensity of the pixels in the images. The transducer was placed perpendicular to the surface of the muscles under examination and adjusted to get the brightest image.

M. supraspinatus: to determine the sites for measuring, the distance between the lateral margin of the acromion and the medial margin of the scapulae at the level of the spina scapulae was measured with a ruler, and a line was drawn parallel to and 2 cm cranial to the spina scapulae. Three measuring sites were marked. The middle site was defined as the midpoint of the drawn line. The medial and lateral sites were located on the line 2 cm medially and laterally to the middle measuring site, respectively, both on the right and left supraspinatus muscles. For each of the supraspinatus muscles, the scanning session consisted of measurements at each of the three measuring sites, longitudinally as well as transversely [19].

M. vastus lateralis: five measuring sites were marked on the right m. vastus lateralis. The middle measuring site was marked in the longitudinal direction by selecting the midpoint between trochanter major and epicondylus lateralis, and in the transverse direction by selecting the midpoint based on muscle palpation. The measuring sites proximal, distal, medial, and lateral, were located 2 cm proximally, distally, medially, and laterally to the middle measuring site.

### Grey scale analysis

**Figure 1:**
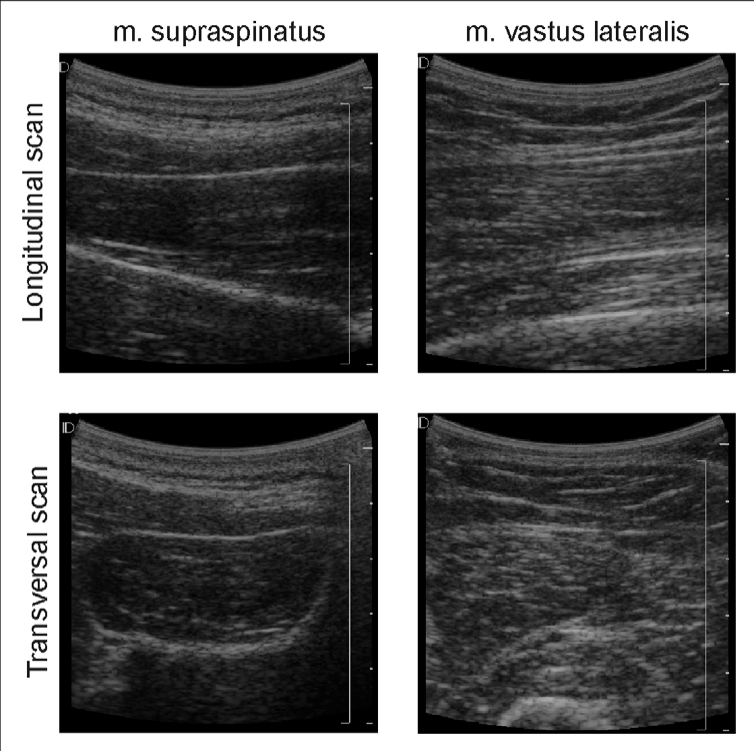
**Ultrasound images of m. supraspinatus and m. vastus lateralis**. Typical ultrasound images of m. supraspinatus and m. vastus lateralis, longitudinal and transversal scanning direction.

Each image consisted of 512 × 512 pixels with grey-scale values ranging from 0 to 255. The lowest values corresponded to the darkest, echo-poor areas in the images, while the highest values corresponded to the brightest, high-intensity areas (Figure 1). The region of interest (ROI) for calculations was defined and marked on each image by visual inspection. The criteria for ROI selection were to include as much of the muscle as possible, and to exclude the surrounding bone and the fascia. Furthermore, any areas indicating poor contact between the probe and the skin were excluded. The range of the ROI size was 10085 to 56893 pixels for the supraspinatus muscles, and 25058 to 93957 pixels for the vastus lateralis muscles mainly depending on the scanning direction.

To evaluate the ultrasound images, two types of computerized image texture analyses programmed in Interactive Data Language (IDL) were used (Interactive Data Language, Research Systems, Inc., Boulder, Colorado, USA). The two analyses derive properties of the image content that are related to intensity, and spatial structure (blob analysis). The blob analysis was developed for the present study. The two types of analyses reflect our hypothesis that the muscle tissue composition can be described by a combination of intensity and structure measures.

The intensity analysis consisted of a computation of the frequency distribution of the grey-scale values of the pixels inside the ROI. All images were filtered by the use of a median filter (kernel size = 3 × 3 pixels) to remove single pixel noise. The reproducibility of this analysis has been described earlier [4].

**Figure 2:**
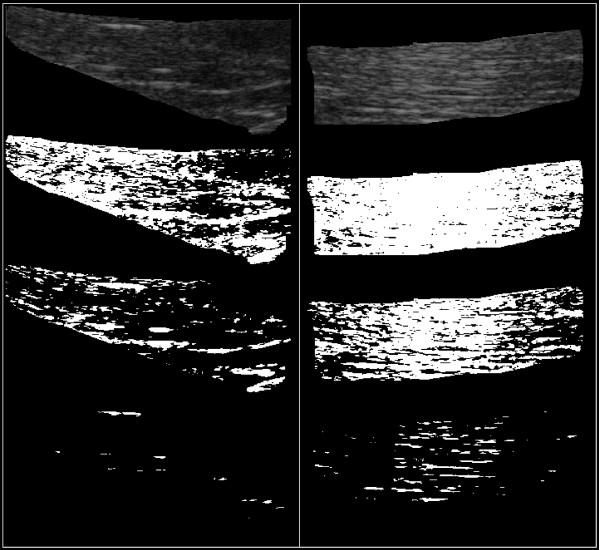
**Regions of interest**. Regions of interest (ROI) of typical ultrasound images of m. supraspinatus (upper left) and m. vastus lateralis (upper right). Binary images at three different thresholds (T). T = 33, (second from the top), T = 53 (second from the bottom) and T = 98 (at the bottom).

The higher-order grey-scale statistical blob analysis consisted of a computation of the number of structures in the image and their size distribution. A simple method of extracting or segmenting the structures in the image is based on thresholding. Thresholding is the transformation of an input grey-level image to an (segmented) output binary image where input pixels with grey-values below the threshold T have been set to 0, and pixels with grey-value above T have been set to 1. In the resulting binary blob image, connected regions consisting of at least three pixels (corresponding to an area of 0.05 mm^2^) with pixel value equal to 1, are referred to as blobs. Parameters like the number of blobs and the size distribution of blobs in the blob image may be computed by counting connected pixels with pixel value 1. Since the structures under interest cannot be segmented by applying a single threshold, we computed blob parameters at several (m) thresholds T. (Figure 2 illustrates the procedure). Two ranges of thresholds were selected, the first range corresponding to the grey-level values of muscle tissue, and the second range corresponding to non-contractile tissue; both previously found in healthy participants [4]. The threshold values were defined on the basis of the grey-scale values in the 25%–75% quartiles interval for the mean grey-scale intensity for the healthy group. The threshold values for muscle tissue were 28–38 and 47–59 for the supraspinatus and the vastus lateralis muscle, respectively. Threshold values for non-contractile tissue were 90–106 in grey-scale intensity [4]. The results presented are the calculated average values of the *number *and the *mean area *of the blobs in these intervals.

Mean blob size, **s**, in the blob image at threshold T (1); Mean number of blobs, **N**, for a set of blob images computed at a range of thresholds (2); and Mean blob size over a range of thresholds, **S, **(3) was calculated as follows:

s=1n∑j=1npj     (1)
 MathType@MTEF@5@5@+=feaafiart1ev1aaatCvAUfKttLearuWrP9MDH5MBPbIqV92AaeXatLxBI9gBaebbnrfifHhDYfgasaacH8akY=wiFfYdH8Gipec8Eeeu0xXdbba9frFj0=OqFfea0dXdd9vqai=hGuQ8kuc9pgc9s8qqaq=dirpe0xb9q8qiLsFr0=vr0=vr0dc8meaabaqaciaacaGaaeqabaqabeGadaaakeaacqWGZbWCcqGH9aqpdaWcaaqaaiabigdaXaqaaiabd6gaUbaadaaeWbqaaiabdchaWnaaBaaaleaacqWGQbGAaeqaaaqaaiabdQgaQjabg2da9iabigdaXaqaaiabd6gaUbqdcqGHris5aOGaaCzcaiaaxMaadaqadaqaaiabigdaXaGaayjkaiaawMcaaaaa@3F2E@

N=1m∑i=1mni     (2)
 MathType@MTEF@5@5@+=feaafiart1ev1aaatCvAUfKttLearuWrP9MDH5MBPbIqV92AaeXatLxBI9gBaebbnrfifHhDYfgasaacH8akY=wiFfYdH8Gipec8Eeeu0xXdbba9frFj0=OqFfea0dXdd9vqai=hGuQ8kuc9pgc9s8qqaq=dirpe0xb9q8qiLsFr0=vr0=vr0dc8meaabaqaciaacaGaaeqabaqabeGadaaakeaaieaacqWFobGtcqGH9aqpdaWcaaqaaiabigdaXaqaaiabd2gaTbaadaaeWbqaaiabd6gaUnaaBaaaleaacqWGPbqAaeqaaaqaaiabdMgaPjabg2da9iabigdaXaqaaiabd2gaTbqdcqGHris5aOGaaCzcaiaaxMaadaqadaqaaiabikdaYaGaayjkaiaawMcaaaaa@3EDF@

S=1m∑i=1msi     (3)
 MathType@MTEF@5@5@+=feaafiart1ev1aaatCvAUfKttLearuWrP9MDH5MBPbIqV92AaeXatLxBI9gBaebbnrfifHhDYfgasaacH8akY=wiFfYdH8Gipec8Eeeu0xXdbba9frFj0=OqFfea0dXdd9vqai=hGuQ8kuc9pgc9s8qqaq=dirpe0xb9q8qiLsFr0=vr0=vr0dc8meaabaqaciaacaGaaeqabaqabeGadaaakeaaieaacqWFtbWucqGH9aqpdaWcaaqaaiabigdaXaqaaiabd2gaTbaadaaeWbqaaiabdohaZnaaBaaaleaacqWGPbqAaeqaaaqaaiabdMgaPjabg2da9iabigdaXaqaaiabd2gaTbqdcqGHris5aOGaaCzcaiaaxMaadaqadaqaaiabiodaZaGaayjkaiaawMcaaaaa@3EF5@

where

***p***_*j *_is the size of blob number ***j ***in the blob image,

***n ***is the number of blobs,

***n***_*i *_is the number of blobs in the blob image at threshold ***i***,

***m ***is the number of thresholds in the range, and

***s***_*i *_is the mean blob size at threshold ***i***.

### Statistics

Data are presented as group mean of 9 participants. In the tables average values, (range), and [quartiles] are used for the average values of the 9 participants, whereas the mean, the variance, the skewness, the kurtosis, and the median are used to describe the distribution of the pixels in the individual ROI-images.

Data was tested for normality, and non-parametric statistics in terms of the Friedman and Wilcoxon-tests were used. The level of significance was selected at p < 0.05 [20]. The Friedman test was used when comparing different measuring sites, and the Wilcoxon test was used when comparing muscles. The Minitab Statistical Software – Release 14 (Minitab Inc, State College, PA, USA).

## Results

### Grey scale intensity

Significant differences in the grey-scale intensities were found between the vastus lateralis muscle and the supraspinatus muscles, as the vastus lateralis was brighter than the supraspinatus (Tables 1 and 2). The measuring sites of the supraspinatus muscles differed significantly and systematically (Table 1), but no significant differences were found with respect to the sites in the vastus lateralis.

The intensities for the supraspinatus muscle from the shoulder patient appeared higher than from the healthy participants, i.e. exceeding the 25–75% quartiles. Similar deviations were also found for the vastus lateralis muscle (Tables 1 and 2).

### Grey-scale structure (blob analysis)

**Figure 3:**
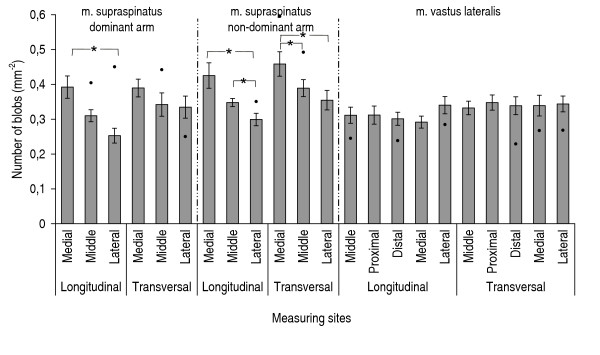
**Number of blobs at grey-scale level typical for muscle tissue**. Number of blobs at grey-scale level typical for muscle tissue for m. supraspinatus right, m. supraspinatus left, and m. vastus lateralis, n = 9. Mean values (S.E.). "●" indicates results from the shoulder patient, when these results exceeded the 25%–75% quartiles for the healthy group. "*" indicates significant differences (p < 0.05) between measuring sites.

**Figure 4:**
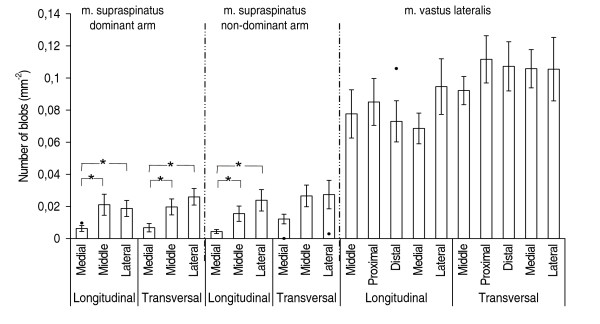
**Number of blobs at grey-scale level typical for non-contractile tissue**. Number of blobs at grey-scale level typical for non-contractile tissue for m. supraspinatus right, m. supraspinatus left, and m. vastus lateralis, n = 9. Mean values (S.E.). "●" indicates results from the shoulder patient, when these results exceeded the 25%–75% quartiles for the healthy group. "*" indicates significant differences (p < 0.05) between measuring sites.

**Figure 5:**
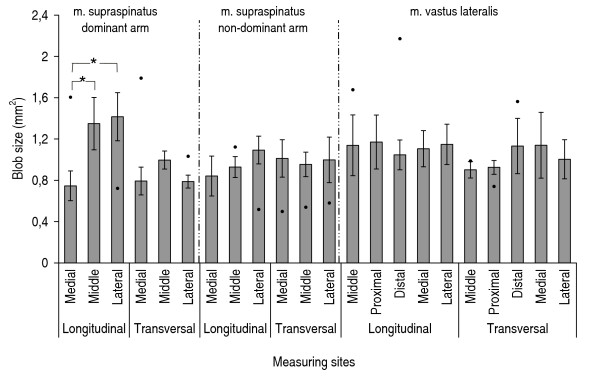
**Mean area of blobs at grey-scale level typical for muscle tissue**. Mean area of blobs at grey-scale level typical for muscle tissue for m. supraspinatus right, m. supraspinatus left, and m. vastus lateralis, n = 9. Mean values (S.E.). "●" indicates results from the shoulder patient, when these results exceeded the 25%–75% quartiles for the healthy group. "*" indicates significant differences (p < 0.05) between measuring sites.

**Figure 6:**
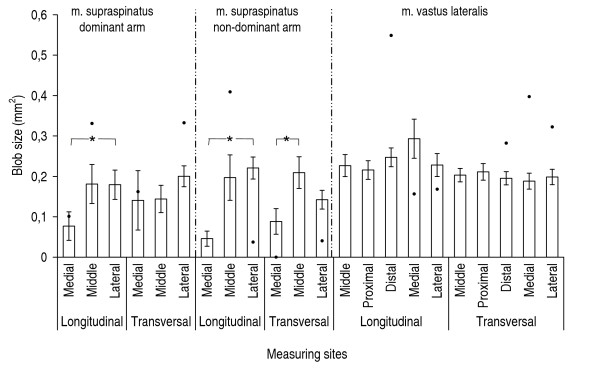
**Mean area of blobs at grey-scale level typical for non-contractile tissue**. Mean area of blobs at grey-scale level typical for non-contractile tissue for m. supraspinatus right, m. supraspinatus left, and m. vastus lateralis, n = 9. Mean values (S.E.). "●" indicates results from the shoulder patient, when these results exceeded the 25%–75% quartiles for the healthy group. "*" indicates significant differences (p < 0.05) between measuring sites.

For the supraspinatus muscle, the number and size of blobs differed significantly between the measuring sites (Figures 3, 4, 5, 6). These differences were more marked in the dominant side. There were no statistically significant differences between the measuring sites for vastus lateralis either at the low-intensity level or at the high-intensity level. The number of blobs at the high-intensity level corresponding to non-contractile tissue differed significantly between the vastus lateralis and the supraspinatus muscles (Figure 4), as the vastus lateralis had more blobs than the supraspinatus. Also the blob size tended to be larger (0.05 < p < 0.1) in the vastus lateralis than in the supraspinatus at the high-intensity level corresponding to non-contractile tissue (Figure 6). No significant differences between muscles were observed for blobs at the low-intensity level corresponding to muscle tissue.

Comparing the shoulder patient with the healthy participants the supraspinatus muscle from the patient had generally more and larger blobs, i.e. exceeding the 25–75% quartiles (Figures 3, 4, 5, 6).

## Discussion

Corresponding to the anatomical structure and composition each muscle seems to have its own specific image defined by the grey-scale intensity and structure of the muscle based on by the computerized ultrasonic B-scan texture analysis. Such quantitative ultrasound is also a potential examination method to detect possible changes associated with use, age and pathology in whole muscles [16] where invasive biopsy technique is not suitable, and for screening purposes or supplementary diagnostic procedures.

In this study the gain settings etc were standardized for all the participants and were kept constant during all measurements to avoid differences in the images due to different setttings of the ultrasound scanner. It is evident that different gain settings and transducers as well as different postprocessing of the images will all influence the absolute grey-scale values. Therefore, so far, each laboratory needs to compile their own reference values. In the future it would be preferable to develop image analyses with a higher level of independency of the equipment.

Furthermore, it is important to avoid undue hard pressure on the imaged tissue since pressure from the transducer may influence the grey scale intensity of the pixels in the images due to changes in the muscle shape and thereby minor changes in the insonation angles. In the present study care was taken that only little pressure was applied on the muscles. As the difference in grey-scale intensity between the shoulder and the thigh muscle was far larger than the potential small difference in grey-scale intensity associated with the pressure exerted on the muscle during scanning we do not think this had blurred the conclusion.

Thus, being aware of these limitations in the grey-scale analyses, an increased proportion of non-contractile tissue might be expected in musculoskeletal disorders [21-24]. Accordingly, the affected supraspinatus muscle differed markedly from the non-affected side and the muscles from the healthy participants, with reservations for the interpretation since only one patient participated. However, some differences could also be an effect of ageing where muscle fibres are often replaced by fat and connective tissue [25] as differences were also present between the patient's non-affected supraspinatus muscle and the vastus lateralis and the muscles in the healthy participants.

### Grey-scale intensity

The grey-scale intensity of the vastus lateralis muscle was higher than the intensity of the supraspinatus muscle, indicating that vastus lateralis contained more non-contractile components than supraspinatus. This result is in accordance with the general anatomy of the two muscles [26]. The vastus lateralis is a long muscle forming the major part of the largest muscle in the body, the quadriceps femoris, and has two large aponeuroses and a tendon. In contrast the supraspinatus muscle is small and triangular with a short tendinous structure. However, in other studies [14,15,27] using a similar method an increased echo intensity of the vastus lateralis muscle was assumed to reflect an increased relative proportion of non-contractile tissue. In the liver Layer et al. [28] demonstrated that connective tissue only lead to a weak increase in the grey-level intensity, whereas the addition of connective tissue to a given tissue lipid might reduce the image brightness. Thus the intensity is so far not able to distinguish between different textures of fat and connective tissue and whether an increased brightness of the images is associated with fat or connective tissue [4].

We found differences between the measuring sites of the supraspinatus muscle, indicating inhomogeneity of the muscle. This is in accordance with previous anatomical findings [29]. Therefore more than one measuring site is needed to describe the muscle as a whole. No differences were found between measuring sites in vastus lateralis, but this fact does not necessarily indicate that the muscle is homogeneous as a whole. The thigh muscle is far larger than the shoulder muscles, and the five measuring sites were concentrated on the middle of the muscle.

### Grey-scale structure

Since the first-order statistics expresses the distribution of grey-scale values without taking their spatial arrangement into account, muscles with identical echogenicity may have very different tissue structure. Therefore, to improve the fidelity of the image analysis we developed the higher-order grey-scale statistics blob analysis, reflecting the structures in the images, to obtain a more complete description of the scanned muscles. Theoretically, a muscle with a significant tendon structure will result in few and large blobs, whereas a muscle with a diffuse structure will result in many small blobs. This means that the blob analysis may quantitatively separate scans of different muscle composition, even though the mean grey-scale intensity is the same.

The number of the blobs was higher and the size of the blobs tended to be larger in the vastus lateralis muscle compared to the supraspinatus muscle at the level corresponding to non-contractile tissue. These findings indicate as expected from anatomical descriptions that the structures in the vastus lateralis muscle are coarser and larger (Figure 1).

## Conclusion

Not only the content of the non-contractile components but also the structure of the muscle are indicated with the newly developed computerized, blob texture analysis, based on the spatial distribution of the grey-scale intensities of the ultrasonic B-scan. Thus, the supraspinatus and the vastus lateralis muscle in the present study seemed to have both different muscle compositions and structures corresponding to their known anatomy. In combination the two quantitative analyses presented, i.e. of the grey-scale intensity and the grey-scale structure (blobs), supplemented each other and gave a more complete description of the tissue composition in the muscle than the mean grey-scale value alone.

## Competing interests

The author(s) declare that they have no competing interests.

## Authors' contributions

All authors conceived of the study, and contributed to the scientific idea and the formulation of the study. All authors participated in choice of technique and experimental design. PKN carried out the ultrasound scanning and performed the statistics. PKN and TD performed the data analysis. All authors participated in the discussion and helped to draft the manuscript. All authors read and approved the final manuscript.

## Pre-publication history

The pre-publication history for this paper can be accessed here:



## Figures and Tables

**Table 1:** First-order grey-scale statistics of m. supraspinatus. M. supraspinatus, dominant arm (D) and non-dominant arm (ND) for n = 9 healthy participants. Average values, (range), and [quartiles] are used for the average value of the 9 participants, whereas the mean, the variance, the skewness, the kurtosis, and the median are used to describe the distribution of the pixels in the individual ROI-images.

**Grey-scale variables**	**Longitudinal scans**						**Transversal scans**					
	
	Medial		Middle		Lateral		Medial		Middle		Lateral	
	
	D	ND	D	ND	D	ND	D	ND	D	ND	D	ND
												
**Mean**	**32.5**	**34.2**	**36.2**	**33.9**	**33.8**	**33.5**	**31.6**	**36.6**	**34.5**	**35.2**	**31.0**	**33.6**
	(18.7–44.2)	(22.4–48.9)	(22.5–47.6)	(27.9–39.7)	(20.0–47.3)	(25.9–45.0)	(25.5–38.5)	(27.5–43.8)	(19.4–42.6)	(27.8–44.4)	(17.6–39.0)	(22.8–47.9)
	[28.6–38.2]	[29.6–39.1]	[32.0–40.9]	[30.4–36.5]	[27.3–39.6]	[28.1–37.7]	[27.6–37.2]	[32.4–41.4]	[30.7–40.1]	[31.2–39.5]	[27.0–35.4]	[30.8–36.8]
												
**Variance**	**167.0**	**165.0**	**250.2**	**252.3**	**284.9**	**293.8**	**190.3**	**191.1**	**246.7**	**256.9**	**282.7**	**258.7**
	(116.0–213.5)	(114.0–263.0)	(128.1–403.8)	(180.9–362.4)	(207.8–363.1)	(175.8–398.1)	(106.9–354.8)	(149.2–250.1)	(175.6–345.6)	(193.1–366.1)	(182.8–380.0	(22.8–47.9)
	[133.3–200.0]	[131.5–198.0]	[217.6–280.7]	[197.2–302.3]	[236.6–334.7]	[235.7–365.4]	[139.4–238.6]	[163.2–220.6]	[184.9–287.6]	[208.3–322.7]	[232.2–336.2]	[30.8–36.8]
												
**Skewness**	**1.1**	**1.0**	**1.2**	**1.4**	**1.2**	**1.3**	**1.2**	**1.2**	**1.3**	**1.4**	**1.6**	**1.3**
	(0.6–1.6)	(0.6–2.1)	(0.7–1.7)	(0.9–2.3)	(0.8–1.7)	(1.1–1.7)	(0.6–2.1)	(0.8–1.7)	(0.7–1.9)	(1.0–1.9)	(1.2–2.0)	(0.6–2.1)
	[0.9–1.3]	[0.7–1.1]	[1.1–1.5]	[1.0–1.6]	[1.0–1.6]	[1.2–1.5]	[0.8–1.4]	[1.0–1.2]	[1.0–1.5]	[1.1–1.8]	[1.5–1.9]	[1.0–1.6]
												
**Kurtosis**	**2.0**	**1.6**	**2.3**	**2.6**	**1.9**	**2.3**	**2.3**	**2.1**	**2.3**	**3.1**	**3.6**	**2.4**
	(0.4–3.9)	(-0.2–7.2)	(0.4–4.7)	(0.8–6.7)	(0.4–4.3)	(1.3–3.9)	(0.5–6.1)	(0.6–5.4)	(0.4–5.3)	(0.8–5.3)	(1.0–5.8)	(-0.2–6.0)
	[0.9–3.2]	[0.4–2.0]	[1.2–3.4]	[0.9–3.7]	[0.9–3.1]	[1.5–2.9]	[1.0–3.3]	[1.5–2.5]	[0.9–3.3]	[1.5–4.4]	[2.7–4.9]	[1.0–3.5]
												
**Median**	**30.4**	**32.2**	**33.3**	**30.9**	**30.1**	**29.8**	**29.3**	**34.3**	**31.3**	**31.9**	**27.0**	**30.4**
	(17.0–43.0)	(20.0–47.0)	(21.0–44.0)	(25.0–36.0)	(16.0–44.0)	(22.0–41.0)	(23.0–44.0)	(26.0–40.0)	(16.0–39.0)	(25.0–40.0)	(14.0–33.0)	(20.0–44.0)
	[26.5–36.0]	[28.0–37.5]	[28.5–38.0]	[27.5–33.0]	[24.0–35.5]	[25.0–33.5]	[25.5–35.0]	[30.5–39.0]	[28.0–37.0]	[28.0–36.0]	[23.5–31.0]	[27.5–33.0]

**Table 2:** First-order grey-scale statistics of m. vastus lateralis. M. vastus lateralis for n = 9 healthy participants. Average values, (range), and [quartiles] are used for the average value of the 9 participants, whereas the mean, the variance, the skewness, the kurtosis, and the median are used to describe the distribution of the pixels in the individual ROI-images.

**Grey-scale variables**	**Longitudinal scans**					**Transversal scans**				
	
	Middle	Proximal	Distal	Medial	Lateral	Middle	Proximal	Distal	Medial	Lateral
**Mean**	**52.1**	**53.0**	**51.1**	**52.3**	**53.6**	**50.6**	**52.4**	**52.9**	**53.1**	**52.3**
	(39.1–68.2)	(42.0–61.9)	(39.0–61.4)	(37.9–61.1)	(41.1–65.5)	(42.7–57.2)	(42.4–58.5)	(38.5–66.2)	(38.9–69.7)	(39.4-64.2)
	[42.7–60.5]	[44.6–59.9]	[42.9–57.6]	[45.2–58.7]	[44.1–60.5]	[45.5–54.7]	[48.6–57.5]	[46.7–57.5]	[46.2–60.1]	[46.6–60.3]
										
**Variance**	**381.6**	**410.4**	**403.7**	**414.4**	**399.1**	**403.4**	**418.0**	**396.8**	**394.8**	**397.7**
	(299.3–513.5)	(331.7–503.2)	(3227–487.0)	(314.6–543.2)	(294.7–566.9)	(361.0–473.3)	(327.3–480.5)	(320.6–501.1)	(330.7–486.2)	(301.7–521.0)
	[326.3–400.8]	[363.5–453.4]	[358.2–447.3]	[366.9–456.8]	[350.8–438.5]	[366.4–435.5]	[393.6–456.4]	[368.8–425.1]	[360.7–424.5]	[326.8–460.2]
										
**Skewness**	**0.7**	**0.7**	**0.8**	**0.8**	**0.6**	**0.9**	**0.8**	**0.7**	**0.7**	**0.8**
	(0.2–1.3)	(0.3–1.1)	(0.4–1.0)	(0.5–1.1)	(0.4–1.0)	(0.5–1.1)	(0.6–1.3)	(0.4–1.1)	(0.3–1.1)	(0.5–1.2)
	[0.5–1.0]	[0.4–1.1]	[0.6–0.9]	[0.6–1.0]	[0.4–0.8]	[0.7–1.0]	[0.6–1.1]	[0.5–1.0]	[0.6–0.9]	[0.6–1.0]
										
**Kurtosis**	**0.3**	**0.3**	**0.4**	**0.4**	**0.2**	**0.5**	**0.4**	**0.3**	**0.3**	**0.5**
	(-0.6–1.8)	(-0.7–1.3)	(-0.3–1.0)	(-0.2–1.6)	(-0.4–1.2)	(-0.2–1.1)	(-0.2–1.6)	(-0.3–1.2)	(-0.6–1.2)	(-0.2–1.7)
	[-0.3–1.1]	[-0.4–1.1]	[-0.2–0.9]	[-0.1–1.0]	[-0.4–0.6]	[0.1–0.8]	[-0.1–1.0]	[-0.1–0.9]	[0.0–0.7]	[-0.1–1.0]
										
**Median**	**49.4**	**49.9**	**47.8**	**49.0**	**50.8**	**46.6**	**48.3**	**49.7**	**49.9**	**49.1**
	(36.0–68.0)	(37.0–61.0)	(36.0–59.0)	(35.0–57.0)	(38.0–64.0)	(37.0–54.0)	(37.0–54.0)	(34.0–65.0)	(35.0–68.0)	(35.0–62.0)
	[39.25–58.5]	[41.0–57.8]	[38.8–53.8]	[41.3–56.0]	[40.3–57.8]	[41.0–51.0]	[44.0–54.0]	[42.5–54.5]	[43.0–57.0]	[42.5–57.5]
